# A benchmark study on current GWAS models in admixed populations

**DOI:** 10.1093/bib/bbad437

**Published:** 2023-11-30

**Authors:** Zikun Yang, Basilio Cieza, Dolly Reyes-Dumeyer, Rosa Montesinos, Marcio Soto-Añari, Nilton Custodio, Giuseppe Tosto

**Affiliations:** Taub Institute for Research on Alzheimer’s Disease and the Aging Brain, College of Physicians and Surgeons, Columbia University, 630 West 168th Street, New York, NY 10032, USA; The Gertrude H. Sergievsky Center, College of Physicians and Surgeons, Columbia University, 630 West 168th Street, New York, NY 10032, USA; Taub Institute for Research on Alzheimer’s Disease and the Aging Brain, College of Physicians and Surgeons, Columbia University, 630 West 168th Street, New York, NY 10032, USA; The Gertrude H. Sergievsky Center, College of Physicians and Surgeons, Columbia University, 630 West 168th Street, New York, NY 10032, USA; Taub Institute for Research on Alzheimer’s Disease and the Aging Brain, College of Physicians and Surgeons, Columbia University, 630 West 168th Street, New York, NY 10032, USA; The Gertrude H. Sergievsky Center, College of Physicians and Surgeons, Columbia University, 630 West 168th Street, New York, NY 10032, USA; Department of Neurology, College of Physicians and Surgeons, Columbia University and the New York Presbyterian Hospital, 710 West 168th Street, New York, NY 10032, USA; Unidad de diagnóstico de deterioro cognitivo y prevención de demencia, Instituto Peruano de Neurociencias, Lima, Perú; Instituto de Neurociencia Cognitiva, Arequipa, Perú; Laboratorio de Neurociencia, Universidad Católica San Pablo, Arequipa, Perú; Unidad de diagnóstico de deterioro cognitivo y prevención de demencia, Instituto Peruano de Neurociencias, Lima, Perú; Taub Institute for Research on Alzheimer’s Disease and the Aging Brain, College of Physicians and Surgeons, Columbia University, 630 West 168th Street, New York, NY 10032, USA; The Gertrude H. Sergievsky Center, College of Physicians and Surgeons, Columbia University, 630 West 168th Street, New York, NY 10032, USA; Department of Neurology, College of Physicians and Surgeons, Columbia University and the New York Presbyterian Hospital, 710 West 168th Street, New York, NY 10032, USA

**Keywords:** GWAS, admixture, benchmark

## Abstract

**Objective:**

The performances of popular genome-wide association study (GWAS) models have not been examined yet in a consistent manner under the scenario of genetic admixture, which introduces several challenging aspects: heterogeneity of minor allele frequency (MAF), wide spectrum of case–control ratio, varying effect sizes, etc.

**Methods:**

We generated a cohort of synthetic individuals (*N* = 19 234) that simulates (i) a large sample size; (ii) two-way admixture (Native American and European ancestry) and (iii) a binary phenotype. We then benchmarked three popular GWAS tools [generalized linear mixed model associated test (GMMAT), scalable and accurate implementation of generalized mixed model (SAIGE) and Tractor] by computing inflation factors and power calculations under different MAFs, case–control ratios, sample sizes and varying ancestry proportions. We also employed a cohort of Peruvians (*N* = 249) to further examine the performances of the testing models on (i) real genetic and phenotype data and (ii) small sample sizes.

**Results:**

In the synthetic cohort, SAIGE performed better than GMMAT and Tractor in terms of type-I error rate, especially under severe unbalanced case–control ratio. On the contrary, power analysis identified Tractor as the best method to pinpoint ancestry-specific causal variants but showed decreased power when the effect size displayed limited heterogeneity between ancestries. In the Peruvian cohort, only Tractor identified two suggestive loci (*P*-value $\le 1\ast{10}^{-5}$) associated with Native American ancestry.

**Discussion:**

The current study illustrates best practice and limitations for available GWAS tools under the scenario of genetic admixture. Incorporating local ancestry in GWAS analyses boosts power, although careful consideration of complex scenarios (small sample sizes, imbalance case–control ratio, MAF heterogeneity) is needed.

## INTRODUCTION

Genome-wide association studies (GWAS) have successfully identified risk and protective loci in many complex human traits. Among these, binary traits have dominated the pool of explored outcomes, e.g. type 2 diabetes or Alzheimer’s disease (AD). Linear mixed models (LMM), extensively used in GWAS with binary traits, violate the assumption of constant residual variance, leading to inflated type I error. The generalized LMM associated test (GMMAT) [1] builds logistic mixed models and constructs a score test for the binary traits in GWAS while accounting for population stratification and relatedness via a kinship matrix. Although GMMAT has been shown to be more robust than other LMM approaches with well-controlled type I error rates, it did not address other common limitations, such as imbalanced case–control ratios, a common scenario in the GWAS—especially in population-based studies where affected cases are usually far rarer than controls. Other limitations, such as rare variants, also lead to *P*-value inflation. To address such limitations, Zhou *et al*. [2] proposed the scalable and accurate implementation of generalized mixed model (SAIGE), which includes Saddlepoint approximation (SPA) [3] in the fitting of generalized LMM, in order to calibrate the score test accounting for imbalanced case–control ratios and rare variants. Through simulation study and real-data analysis, SAIGE shows well-calibrated *P*-values even under these extreme scenarios.

Another pressing issue in GWAS is the under-representation of admixed populations, whose genomes contain segments inherited from multiple ancestral groups. Few GWAS tools have been specifically designed for such complex genetic architecture. Tractor [4] is a scalable framework that incorporates the genetic structure of admixed individuals into large-scale genomics efforts through local ancestry inference, which has been shown to be capable of detecting and modeling ancestry-specific effect sizes. The impact of the local ancestry on association models has also been investigated in a recent publication [5], where the authors compared the performances of Tractor versus other methods based on the Armitage trend test. However, the latter are fixed-effect models that do not consider random effects such as the genetic relatedness between individuals. The paper also did not account for imbalanced case control ratio, rare variants, etc. Therefore, the performances of Tractor have yet to be systematically examined to their full extent.

While there have been previous benchmarking efforts [6, 7], these studies primarily focused on the challenges posed by rare variants or heterogeneous studies without considering the complexities introduced by genetic admixture. In general, there is a lack of standardized criterion for benchmarking popular GWAS methods and their results under a variety of key factors, such as minor allele frequency (MAF) heterogeneity, imbalanced case–control ratio, admixture, etc. In this study, we present a benchmark investigation that fills the gap by systematically examining the performances of three popular GWAS models, GMMAT, SAIGE and Tractor, conditional on the factors stated previously. We also applied these tools in an AD study of admixed Peruvians from the ‘Genetics of Alzheimer’s disease In Peruvian Populations study’ (GAPP) study.

## METHODS

### Data process

We employed a large synthetic dataset using HAPNEST [8], a recently developed software that enabled the generation of a diverse synthetic datasets (using publicly available reference datasets) of 1 008 000 individuals from six different ancestral groups. We used the Admixed American (AMR) group from HAPNEST and performed phasing using the 1000 Genome project [9] (1000G) as reference haplotype panel and *Shapeit* [10] (2.r837). We then used *RFMix2* [11] (v2.0.3), a discriminative approach that estimates both global and local ancestry using random forests, to inference global ancestry assuming a three-way admixed scenario, i.e. Native-American (NAA), European (EUR) and African (AFR) ancestry, employing the Human Genome Diversity project (HGDP) [12] as reference panel. We then filtered out individuals with significant African global ancestry (i.e. the African global ancestry ≥ 10%) in order to retain a two-way admixed sample. We again used *RFMix2* to estimate local ancestry assuming a two-way admixture (NAA-EUR) on the remaining 19 234 individuals. We used 19 081 independent genetic variants on Chromosome 20, limiting the analyses to variants with minor allele count (MAC) > 10 to investigate the performances of the testing methods along the whole spectrum of allele frequency (i.e. from ultra-rare to common causal variants). The major ancestry of the two-way admixed individuals simulated in HAPNEST is EUR, and the minor ancestry is NAA.

To apply our methods and perform real-data analyses, we leveraged the GAPP study, a recently established cohort of Peruvian mestizos from Lima and indigenous groups from Southern Peru (Aymaras and Quechuas). Genotyping was conducted on the Infinium Global Screening Array-24 BeadChip, which combines multi-ethnic genome-wide content, curated clinical research variants and quality control (QC) markers for precision medicine research, extensively detailed in previous publications from our group. Ultimately, our GWAS analysis incorporated a total number of 5 279 846 variants. We conducted the same procedures as previously described, by first phasing the genetic data, then estimating global ancestry and finally (after excluding three individuals with high African global ancestry) inferencing local ancestry assuming a two-way admixture (NAA and EUR) on the remaining 249 individuals. Variants were filtered out if MAC < 5.

### Simulation setting

We conducted a series of simulations to evaluate the performances of the testing methods under a variety of different scenarios. We evaluated the performances of the testing methods from two perspectives, i.e. the control of type I error rates and the empirical power for detecting the true effect sizes.

(i) Control of type I error. For testing the control of type I error rates, the binary phenotypes were generated by a logistic mixed model,


$$ \mathrm{logit}\left(\mu \right)={\alpha}_0+b+{X}_1+{X}_2+G\ast \beta, $$

where *G* is the genotype, $\beta$ is the genetic log odds ratio and *b* is the random effect simulated from a normal distribution N (0, $\varphi$) with the relatedness matrix $\varphi$. Two covariates, ${X}_1$and ${X}_2$, were drawn from Bernoulli (0.5) and standard normal distribution, which represents the discrete and quantitative predictors. The intercept alpha was chosen to represent the corresponding probability of the disease. Under the scenario of the control of type I error, the phenotypes were simulated with $=0$ . We also simulated three case–control ratios as 1:1, 1:9 and 1:99, denoted as ‘balance’, ‘imbalance’ and ‘extreme-imbalance’ scenarios. We then computed and compared the inflation factor *lambda* for each testing method. We also computed the total numbers of the *P*-values smaller than the genome-wise significance (i.e. *P*-values ≤ 5 * ${10}^{-8}$) to examine the control of the type I error for each testing method.

(ii) Power analysis. In this scenario, we investigate performances of the methods under the scenario of the ancestry-specific effect on the phenotype. Phenotypes were simulated under the alternative hypothesis, i.e. $\beta$ of the causal variant is not equal to 0. To facilitate the admixture scenario, we simulated that the risk allele was only associated with the NAA ancestry. First, we randomly selected a risk variant conditional on the pre-determined thresholds of MAF. Then, we simulated the phenotype through the probability of disease, which is set to,


$$ \mathrm{logit}\left(\mu \right)={\alpha}_0+b+{X}_1+{X}_2+{G}_{\mathrm{NAA}}\ast{\beta}_{\mathrm{NAA}}, $$

#### MAF

We categorized the results of the testing causal variants according to the corresponding MAF, such as ‘ultra-rare’ (MAF < 0.001), ‘rare’ (0.001 < MAF < 0.01), ‘uncommon’ (0.01 < MAF < 0.05) and common (MAF > 0.05).

#### Varying effect size

We simulated 100 replicates of simulated genotypes with a logistic model for each allelic effect sizes of the causal ancestry NAA (${\beta}_{\mathrm{NAA}}$) set at 0.25, 0.5, 0.75, 1.0, 1.5, 2.0, 2.5 and 3.0, whereas the effect size of the null ancestry EUR is 0 (${\beta}_{\mathrm{EUR}}=0$).

#### Case–control ratio

We again considered three case–control ratios: 1:1, 1:9 and 1:99, denoted as ‘balance’, ‘imbalance’ and ‘extreme-imbalance’ scenarios.

(iii) Impact of sample sizes. To assess the effect of sample size on power and false positives, we repeated the analyses described in (ii) with progressively increasing sample sizes: 500, 1000, 2000, 5000 and 10 000. Within each specific scenario of MAF, case–control ratio and sample size, synthetic individuals were sampled from the datasets described in point (ii). Additionally, 100 replicates were generated under each scenario to ensure robustness of the findings. The effect sizes for ‘ultra-rare,’ ‘rare,’ ‘uncommon’ and ‘common’ MAF were set at 3.0, 2.0, 1.0 and 0.5, respectively.

(iv) Impact of heterogeneity of effect sizes between ancestries. To investigate the impact of the heterogeneity of effect sizes when the causal variants have non-zero causal effect sizes in both ancestries, we conduct a secondary analysis assuming ${\beta}_{\mathrm{EUR}}=0.15$ and $-0.50\le{\beta}_{\mathrm{NAA}}\le 0.65$ increasing by 0.05. The case–control ratio was set at 1:3 to represent real-world situation [13], and the MAFs of the causal variants of both ancestries were set between 0.1 and 0.2. We measured the testing methods’ performance through power as defined previously in (ii). We also explored a scenario where each ancestry has distinct causal variants. In this setup, the causal variant for the EUR ancestry remained consistent with our previous simulation, while the causal variant for the NAA ancestry was selected based on the highest linkage disequilibrium (LD) with the EUR ancestry’s causal variant.

### GWAS methods

For simulations described in (i) and (ii), we fitted the three GWAS tools, GMMAT, SAIGE and Tractor. We generated the genomic relatedness matrix (GRM) through PLINK/2.0^1^ and provided it to GMMAT, whereas SAIGE creates a sparse relatedness matrix with a default threshold at 0.125 and Tractor does not include the relatedness matrix in its association test. We included the first three principal components to account for population structure. The simulated covariates, ${X}_1$ and ${X}_2$, were also provided to the testing models. Tractor (version 0.0.1) fits a logistic regression model including the two ancestry-specific genotypes (while accounting for covariates and local ancestry) then returns the estimated ancestry-specific *P*-values (in this experiment, we obtained two statistics for EUR and NAA). When computing the false positives for SAIGE, we excluded variants flagged by the software when the SPA algorithm did not converge. For simulations described in (iii) and (iv), we only fitted and compared results from GMMAT and Tractor. In fact, assuming a case–control ratio = 1:3, and the causal variants set as common variants, we did not observe any difference between GMMAT and SAIGE (data not shown). In simulation (iv), we applied GMMAT to the local ancestry and compared the results with Tractor.

We further compare the run times of the testing methods, where the experiments were conducted on a system with Debian GNU/Linux 10 as its operating system. The machine’s architecture is x86_64, with CPU op-modes for 64-bit. It is powered by an 8-core Intel(R) Xeon(R) CPU E5-2620 v4, operating at a base frequency of 2.10GHz, and frequencies of 1200.392 MHz. For each job executed on this setup, a memory allocation of 20 GB was designated.

For the real-data analysis from GAPP, we again only fitted and compared the performances of GMMAT and Tractor, since the case–control ratio was not simulated but derived from real diagnostic status, i.e. 1:3 (58 cases versus 190 controls). We also restricted our analyses to common variants only. Therefore, GMMAT and SAIGE produced again overlapping results (data not shown). The first three principal components, age and sex were also used as fixed affects and the GRM as random effect. The local ancestry dosage generated by RFMix was used to implement Tractor as described previously.

## RESULTS

### Global ancestries in the synthetic cohort and in GAPP

**Figure 1:**
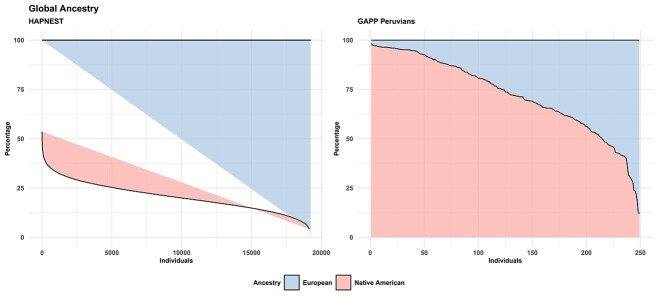
Global ancestries of the individuals included in the HAPNEST and GAPP Peruvians.

Figure 1 shows the global ancestry distribution for the two cohorts employed in this project, i.e. the synthetic admixed cohort from HAPNEST and the Peruvians from GAPP. The major and minor ancestry of the Peruvian cohort are NAA and EUR, respectively, whereas the major and minor ancestry of HAPNEST are EUR and NAA, respectively.

### Type I error rates

**Figure 2:**
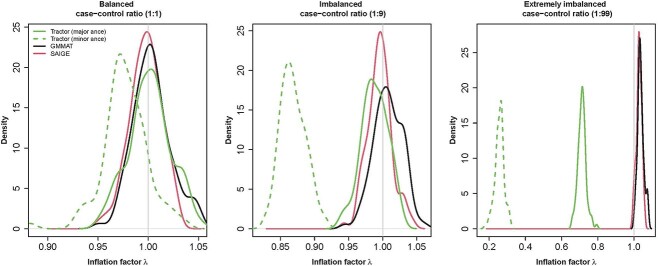
The density function of the inflation factors of the three testing methods over 100 replicates in the simulation scenario of type-I error control.

All three methods attained acceptable inflation factors with a well-balanced case–control ratio (1:1, Figure 2). SAIGE showed well-calibrated inflation factors compared to GMMAT when the case–control ratio shifted to 1:9, whereas Tractor started to show decline in *P*-values calibration, especially for *P*-values associated with the minor local ancestry (i.e. NAA). SAIGE and GMMAT both exhibited small inflation in extremely imbalanced case–control ratio (1:99), whereas Tractor showed problematic *P*-values calibration with severe deflation for both major and minor global ancestries. As shown in Supplementary Table 1, GMMAT and Tractor produced genome-wide significant results when the case–control ratio is extremely unbalanced. False positive rates (FPR) were strongly correlated with low MAFs, with GMMAT’s false positives associated with the ultra-rare variants, whereas Tractor’s false positives extend in the range of rare variants as well. On the other hand, SAIGE was the only method that did not produce false positives under any case–control scenario, and ultimately confirmed the conclusions reached by their authors in its published manuscript. The deflation of Tractor’s *P*-values in Figure 2 is conditional of variants’ MAF (Supplementary Table 2 and Supplementary Figure 1), where variants with *P*-values < 0.99 show MAFs higher than those variants with *P*-values ≥ 0.99.

### Power analysis

**Figure 3:**
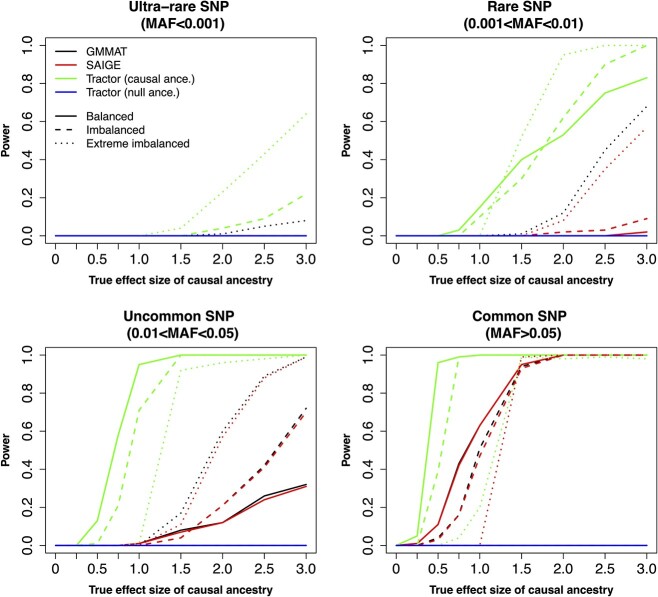
Power calculation of the three methods based on the 19 234 synthetic individuals from HAPNEST. The significance threshold of *P*-value is set at genome-wide significance (*P* < 5e-8). The causal ancestry, i.e. the corresponding effect size is non-zero, is NAA.

Under large sample sizes (such as the HAPNEST cohort, Figure 3), Tractor showed superior performance in terms of power, whereas the performances of GMMAT and SAIGE were virtually similar. For ultra-rare and rare causal variant, Tractor also performed better, although required large true effect sizes. For uncommon or common MAF, Tractor again performed better than GMMAT and SAIGE in identifying causal variants with smaller effect sizes under different scenarios of case–control ratio. Tractor also successfully identified the ‘causal ancestry’ (green line in Figure 3), i.e. the ancestry that harbors the causal variants (non-zero effect sizes).

As reported in Table 1, under a balanced case–control ratio Tractor controls well FPR as we did not observe any false positive results associated with the ‘null ancestry’, i.e. the ancestry that does not harbor the causal variant. However, Tractor retrieved concerningly higher rates of false positives under extremely imbalanced case–control ratio, compared to GMMAT and SAIGE. These false positives were again associated mainly with ultra-rare variants.

**Table 1:** Average number of genome-wide significant variants (‘GWV’, *P*-value $\le 5\ast{10}^{-8}$) (and their median MAF) averaging over all 2400 replicates stratified by case–control ratio. The median MAF for variants identified by Tractor are computed based on local ancestry dosage. We excluded the true causal variants when computing numbers shown in this table

2400 replicates		GMMAT	SAIGE	Tractor	
				Major/Null ancestry	Minor/Causal ancestry
Balanced	# GWV	0.24	0.18	0	0.16
	Median MAF	0.033	0.033	NA	0.14
Imbalanced	# GWV	0.4	0.26	0.032	0.23
	Median MAF	0.04	0.034	0.00078	0.014
Extremely	# GWV	1.18	0.2	2.03	4.93
Imbalanced	Median MAF	0.018	0.047	0.0026	0.0062

### Impact of sample size

Consistent with the power analysis results, Tractor consistently outperformed GMMAT and SAIGE in terms of power by detecting true causal variants even at smaller sample sizes and maintaining substantial higher power as sample size increases. Supplementary Figure 2 illustrates the performance of GMMAT, SAIGE and Tractor under varying sample sizes. Tractor successfully identified the causal ancestry at smaller sample sizes, while SAIGE showed limited ability to identify causal variants unless the sample size exceeded *N* = 10 000. GMMAT exhibited non-zero power in the scenario of ultra-rare causal variants and extremely imbalanced case–control ratios; however, this was attributed to a large number of false positives associated with low MAFs (which included the ultra-rare causal variants). GMMAT and Tractor tend to generate false positives associated with low MAF (Supplementary Table 3); conversely, SAIGE demonstrated robust performances across a range of scenarios, including different sample sizes and case–control ratios, but only when we filtered out a large number of testing variants with non-converge SPA algorithm. Supplementary Table 4 shows that the mean and median of MACs of the testing variants with non-converged SPA algorithm is smaller than the MACs with converged SPA algorithm in SAIGE.

### Heterogeneity of effect sizes between ancestry

**Figure 4:**
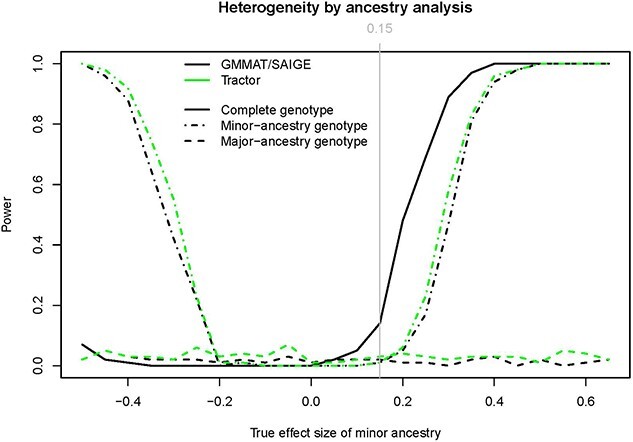
Power calculations for one causal variant with heterogenous effect sizes between the two ancestries (i.e. NAA and EUR). The effect size of causal variant within the minor ancestry (${\beta}_{\mathrm{NAA}}$) ranges from −0.5 to 0.65 by 0.05, whereas the effect size of the causal variant within major ancestry (${\beta}_{\mathrm{EUR}}$) is fixed at 0.15.

GMMAT/SAIGE (which employ the genotype data without any deconvolution by local ancestry), are consistently more powerful than Tractor when the effect sizes of the two ancestries are in same direction, i.e. with limited heterogeneity between major (EUR) and minor (NAA) ancestry’s effect sizes (Figure 4). On the other hand, when the effect sizes of major and minor ancestry are in opposite directions, i.e. ${\beta}_{\mathrm{EUR}}=0.15$ while ${\beta}_{\mathrm{NAA}}<0$, GMMAT/SAIGE display low power due to the cancelation of opposite effect sizes. On the contrary, Tractor picks up the causal variants when, for example, the effect size associated with the minor ancestry is large enough to overcome the opposite effect size associated with the major ancestry. Further, when GMMAT/SAIGE were applied to the ancestry-specific genotype, they exhibited similar performances to the results obtained by Tractor in terms of (i) increased power when the heterogeneity between the effect sizes is large and (ii) reduced power when the heterogeneity is small.

In Supplementary Figure 3, we observe that Tractor still demonstrates greater power with the ancestry-specific genotype matrix compared to traditional GWAS methods when causal variants differ between the two ancestries. The performance of Tractor in this scenario is largely consistent with its performance when the causal variant is identical across ancestries. In contrast, GMMAT’s power is nearly halved compared to the previous scenario. This reduced power is attributed to GMMAT’s difficulty in detecting the small-scale effect size associated with the EUR ancestry. Consequently, the observed power of GMMAT predominantly arises from the detection of causal variants within the NAA ancestry.

### Real-data analysis

**Figure 5:**
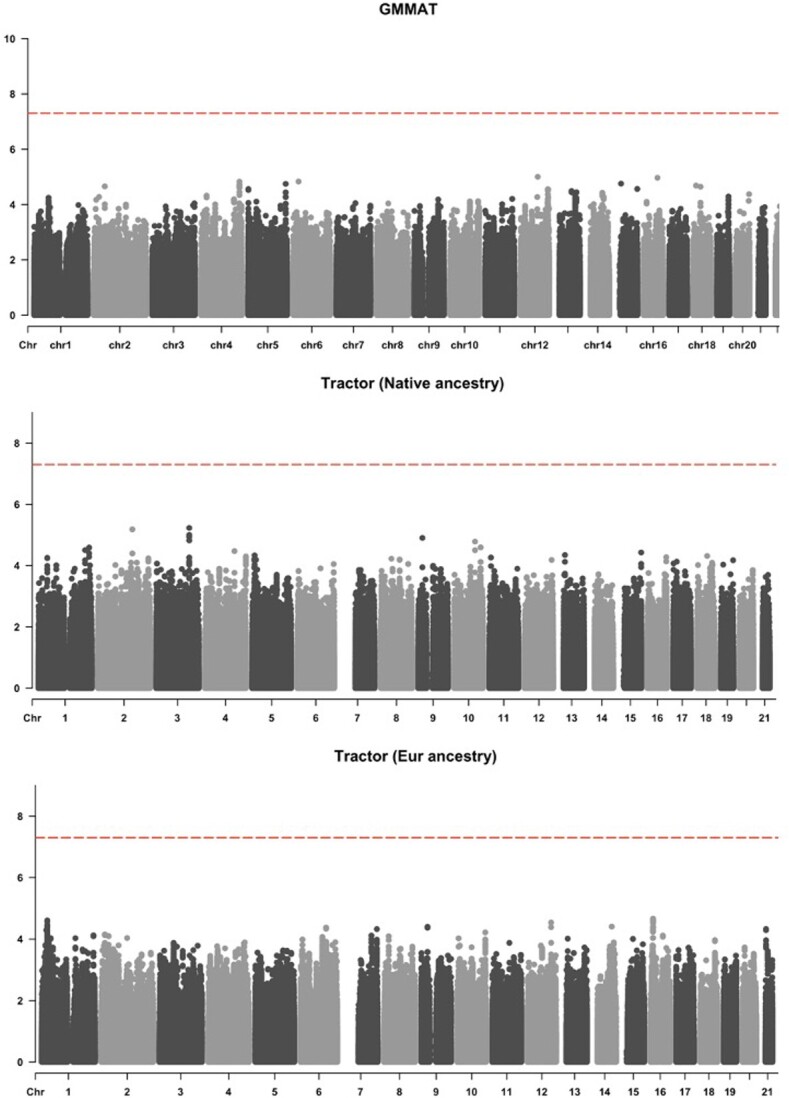
Manhattan plots of AD GWAS produced by GMMAT (**A**) and Tractor (**B**: NAA and **C**: EUR) in the GAPP cohort. The total number of tested variants is 4 492 989.

Figure 5 shows no genome-wide significant results achieved in GAPP using GMMAT and Tractor, likely due to the relatively small sample size (*N* = 249). Nevertheless, only Tractor identified three variants with suggestive significance at *P*-value $\le 1\ast{10}^{-5}$, as shown in Supplementary Table 5. There was no evidence of *P*-value inflation for GMMAT or Tractor (GMMAT: $\lambda$= 0.97; Tractor NAA: $\lambda$= 1.02; Tractor EUR: $\lambda$=1.04).

### Computational time

Supplementary Table 6 indicates that SAIGE outperformed GMMAT and Tractor significantly.

## DISCUSSION

In this study, we employed large and small admixed cohorts of synthetic and real-data individuals, and benchmarked the results obtained by three popular GWAS methods, GMMAT, SAIGE and Tractor under various scenarios.

When studying the type-I error control, SAIGE generated well-calibrated *P*-values even under extreme situations, such as rare variants or imbalanced case–control ratios. GMMAT showed small *P*-values inflation and produced false positives only under extreme scenarios. On the other hand, Tractor showed severe *P*-values deflation under the scenarios associated with extreme case–control ratios, while still producing false positives at global-wise significant level. Therefore, we concluded that Tractor’s calibrations of *P*-values are greatly affected by imbalanced case–control ratios, even when sample sizes are large, particularly for the minor ancestry, which is equivalent to the reduction on sample size. Tractor’s severe deflation observed in the density plot depicted in Figure 2 is likely due to a large proportion of rare variants generated by the deconvolution operated at the local ancestry estimation. The extreme phenotypic variance caused by the extreme case–control ratio could also contribute to the deflated *P*-values, as demonstrated previously [14].

When testing power calculations, we observed optimal performances by Tractor in identifying and modeling the causal effect sizes, compared to the GMMAT and SAIGE. The superiority of Tractor was particularly evident when large heterogeneity existed in terms of effect sizes between ancestries. Supplementary Figure 3 further demonstrates the advantages of applying Tractor on the ancestry-specific genotypes when the causal variants were associated with distinct ancestries. Our conclusion is in line with results reported in the original paper [4] and other recently published benchmark papers, e.g. [5]. In the latter, the authors reported a loss of power of ~70% from Tractor when compared to the traditional GWAS methods. The authors attribute Tractor’s loss of power to (i) differences in ancestry-specific allele frequencies of the causal variants and (ii) the penalty from an additional degree of freedom in the association test (e.g. ${\beta}_{\mathrm{NAA}}=0$ and ${\beta}_{\mathrm{EUR}}=0$ simultaneously) [5]. We replicated this loss of power by simulating two distinct causal variants harboring within the two ancestries (Supplementary Figure 3) as opposed to the scenario where the same causal variant harbors in both ancestries (Figure 4). As for the degree of freedom, the latest version of Tractor provides the marginal test for the effect size of each ancestry with the same degree of freedom of the association test used by GMMAT/SAIGE. Therefore, we conclude that Tractor’s loss of power is likely due to deconvolution of the genotype matrix, which is analogous to reducing the sample size, especially when the heterogeneity across effect sizes is not large enough. This has been clearly depicted in Figure 4 by showing that similar power was reached by GMMAT/SAIGE and Tractor when executed on the ancestry-specific genotype matrix, which indicates that the loss of power should not be solely attributed to the difference in the association tests of the additional degree of freedom.

Given HAPNEST large sample size and balanced case–control ratio, GMMAT and Tractor shows well-controlled FPR, but produced false positive results associated with the rare variants and extreme case–control ratio in both simulation studies (type I error control and power analysis). We confirmed the mishandling of rare variants analyses when we tested the impact of sample size on power calculations: indeed, GMMAT produced a large number of false positives associated with low MAF at small sample sizes (*N* ≤ 1000) and extremely imbalanced case–control ratio, while Tractor reported moderate FPR. SAIGE did not generate false positives across simulated sample sizes, although this optimal performance was achieved only after filtering out a substantially large number of variants flagged by failed convergence of the SPA algorithm (mostly associated with low MAF and MAC under the extreme imbalanced scenarios). In other words, SAIGE can only provide reliable results for common variants under the extreme imbalanced scenario and small sample sizes. As noted in SAIGE’s manual, its performance in small sample size scenarios has not been thoroughly examined, and our finds from the simulation addressed this gap.

Surprisingly, Tractor displayed lower power under balanced case–control ratio compared to (extremely) imbalance case–control ratio for ultra-rare and rare causal variants. This is likely due to the genotype vector of ultra-rare (or rare) causal variant (${G}_{\mathrm{NAA}}$) being a nearly sparse vector and having limited impact on the random sampling of the binary phenotypes under a balanced scenario, which is largely controlled by the intercept that is universal to all subjects. On the other hand, under the (extreme) imbalanced scenario, the small number of cases are mostly associated with the (ultra) rare causal variant with large effect size. This resulted in Tractor showing higher power under (extreme) imbalance case–control and (ultra) rare causal variant scenarios.

It should be noted that Tractor may not be suitable for analyzing related samples, as Tractor does not account for kinship, which could lead to inflated *P*-values/false positives.

SAIGE outperformed the other two methods significantly in terms of run times. This advantage can be attributed in part to SAIGE’s use of the preconditioned conjugate gradient approach, which solves linear systems for extensive cohorts, coupled with its efficient memory utilization during model fitting. In contrast, Tractor lags both methods, primarily because it employs two ancestry-specific genotype matrices and conducts an extra hypothesis test during its model fitting process.

We purposely employed a small real-data study (GAPP) to show the software’s performance under this scenario. Admixed populations are traditionally underrepresented in genetic studies. Similarly to other studies conducted in non-White populations, the size of our Peruvian GWAS is comparable to many other previously published GWASs (with the additional value of this being the first ever GWAS of AD in Peruvians). For example, one of the largest and most cited non-White GWAS for AD [15] is in reality obtained by meta-analyzing 14 independent studies, 12 of which are small (five of those are *N* < 200; Supplementary Table 3). Our results will have an impact not only on small-sized studies, but also on large GWAS derived from meta-analyses of small datasets.

Within the GAPP cohort analysis, despite a limited sample size of *N* = 249, no genome-wide significant results were observed using either GMMAT or Tractor. The absence of *P*-value inflation for both methods (GMMAT: λ = 0.97; Tractor NAA: λ = 1.02; Tractor EUR: λ = 1.04) confirms that both models were well-calibrated when the case–control ratio is balanced. Moreover, Tractor identified three variants associated with the NAA ancestry with suggestive significance, a finding not mirrored by GMMAT. This result in real-data analysis complements the simulation results, where Tractor consistently demonstrated superior power, particularly in balanced scenarios with common variants. While this does not serve as direct validation, it subtly implies a consistent trend in Tractor’s efficacy, both in simulated and real-data contexts. As suggested in [5], in real analysis standard GWAS tools and Tractor often prioritize distinct loci, therefore considering both methodologies could be effective when working with real-world data of admixed population.

In summary, we acknowledge the improvement achieved by Tractor in identifying ancestry-related causal variants, by leveraging the unique genetic structure of admixed populations. However, we want to caution the usage of Tractor under extreme circumstances, especially in small sample sizes and when the deconvolution of genotype matrix introduces additional issues in terms of allele frequency. This study demonstrates the importance of considering imbalanced case–control ratio, rare variants and varying sample size, and ultimately addresses the major challenges for the development of future GWAS methods in admixed populations.

**Key Points**

- We benchmarked the performances of three popular GWAS tools, GMMAT, SAIGE and Tractor, under a variety of key aspects, such as minor allele frequency heterogeneity, effect size of causal variants, imbalanced case–control ratio and admixture status.
- Tractor outperformed GMMAT and SAIGE when there is a significant heterogeneity of effect sizes between ancestries.
- GMMAT produced substantial false positives when case–control ratio is imbalanced and sample size is small, whereas SAIGE is resilient to that scenario. Tractor reported moderate false positive rates.

## Supplementary Material

supplementary_10_25_yzk_bbad437

## Data Availability

The analysis code to produce the major results presented in the paper is available at https://github.com/ZikunY/Benchmark_GWAS. The synthetic data used in the simulation study are available at https://www.ebi.ac.uk/biostudies/studies/S-BSST936. Request for genetic and phenotype data of Peruvian cohort can be submitted to gt2260@cumc.columbia.edu.

## Author Biographies

**Zikun Yang** is a post-doctoral researcher in Biostatistics at Columbia University (NY, USA). His main line of research is developing new methods to effectively analyze complex genetic data from admixed populations.

**Basilio Huaman** is a post-doctoral researcher at Columbia University (NY, USA). He is a bioinformatician working in implementing genome-wide association and sequencing studies in admixed populations.

**Dolly Reyes-Dumeyer** is a senior project coordinator at Columbia University (NY, USA). She overviews several large recruiting efforts within and outside United States to recruit Hispanic/Latino individuals for genetic studies of Alzheimer’s Disease.

**Rosa Montesinos** is the head of Rehabilitation service at the Peruvian Institute of Neurosciences (Lima, Peru). She overviews the operations of several international projects recruiting South American participants for multi-institutional studies.

**Marcio Soto-Añari** is a psychologist and professor of the School of Psychology of the San Pablo Catholic University and the National University of San Agustín de (Arequipa, Peru).

**Nilton Custodio** is the director of the Peruvian Institute of Neurosciences (Lima, Peru). He is a neurologist working on brief cognitive tools, particularly in low-educated and illiterate populations, including Quechua-speaking.

**Giuseppe Tosto** is an assistant professor of Neurology at Columbia University (NY, USA). His lab investigates the genetic architecture of Alzheimer’s disease in admixed populations. He is the Principal Investigator of several projects recruiting Hispanic/Latino participants in United States and South America.
