# Correction to: A benchmark study on current GWAS models in admixed populations

**DOI:** 10.1093/bib/bbae114

**Published:** 2024-03-12

This is a correction to: Zikun Yang, Basilio Cieza, Dolly Reyes-Dumeyer, Rosa Montesinos, Marcio Soto-Añari, Nilton Custodio, Giuseppe Tosto, A benchmark study on current GWAS models in admixed populations, *Briefings in Bioinformatics*, Volume 25, Issue 1, January 2024, bbad437, https://doi.org/10.1093/bib/bbad437

In the originally published version of this article, in Figure 1, the image in the panel entitled HAPNEST was incorrect.

The Publisher apologizes for this.

Figure 1 has been corrected.

