# Supplementary material for: A benchmark study on current GWAS models in admixed populations
Source: Brief Bioinform. 2023 Nov 30;25(1):bbad437. doi: 10.1093/bib/bbad437 (PMC10689347; doi:10.1093/bib/bbad437)
Supplement: supplementary_10_25_yzk_bbad437 [file supplementary_10_25_yzk_bbad437.docx]

**A benchmark study on current GWAS models in admixed populations.**

**SUPPLEMENTARY MATERIALS**

| **Supplementary Table 1...............................................................................................................** | **2** |
| --- | --- |
| **Supplementary Table 2...............................................................................................................** | **3** |
| **Supplementary Table 3...............................................................................................................** | **4** |
| **Supplementary Table 4...............................................................................................................** | **5** |
| **Supplementary Table 5...............................................................................................................** | **6** |
| **Supplementary Table 6...............................................................................................................** | **7** |
| **Supplementary Figure 1..............................................................................................................** | **8** |
| **Supplementary Figure 2..............................................................................................................** | **9** |
| **Supplementary Figure 3..............................................................................................................** | **10** |

**Supplementary Table 1**. Number of genome-wide significant variants (“GWV”, p-value ≤5e-8) over 100 replicates, and the median MAF of these variants under the extremely imbalanced case-control scenario (1:99). Tractor variants’ MAF is computed based on ancestry-specific genotypes.

|  | **GMMAT** | **SAIGE** | **Tractor** | |
| --- | --- | --- | --- | --- |
|  |  |  | Major ancestry | Minor ancestry |
| # GWV | 66 | 0 | 4 | 1 |
| Median MAF | 0.0009 | NA | 0.0072 | 0.0006 |

**Supplementary Table 2.** Variants’ median MAF stratified by p-values as reported by Tractor. Major ancestry is EUR, minor ancestry is NAA.

|  | **Variants with p-values<0.99** | | | **Variants with p-values**$\boldsymbol{\geq}$**0.99** | | |
| --- | --- | --- | --- | --- | --- | --- |
|  | Major ancestry | Minor ancestry | Proportion | Major ancestry | Minor ancestry | Proportion |
| **Balanced (1:1)** | 0.021 | 0.018 | 97.17% | 0.0085 | 0.0074 | 2.83% |
| **Imbalanced (1:9)** | 0.023 | 0.019 | 93.16% | 0.0028 | 0.0022 | 6.84% |
| **Extremely imbalanced (1:99)** | 0.045 | 0.037 | 65.27% | 0.0057 | 0.0051 | 34.73% |

**Supplementary Table 3.** Average number of genome-wide significant variants (“GWV”, p-value $\leq{5*10}^{-8}$) and their median MAF over 400 replicates, stratified by case-control ratio and sample size. The median MAF for variants identified by Tractor are computed based on local ancestry dosage. We excluded the true causal variants when computing numbers shown in this table. “N” represents the sample size of each scenario.

|  | **GMMAT** | | **SAIGE*** | | **Tractor** | | | |
| --- | --- | --- | --- | --- | --- | --- | --- | --- |
|  |  |  |  |  | Major/Null ancestry | | Minor/Causal ancestry | |
|  | # GWV | Median MAF | # GWV | Median MAF | # GWV | Median MAF | # GWV | Median MAF |
| **N=500** | | | | | | | | |
| Balanced (1:1) | 0.0075 | 0.001 | 0 (0.0025) | NA (0.034) | 0.03 | 0.00211 | 0.0325 | 0.00175 |
| Imbalanced (1:9) | 4.9575 | 0.001 | 0 | NA | 0 | NA | 0 | NA |
| Extreme imbalanced (1:99) | 51.64 | 0.005 | 0 (2332.41) | NA (0.061) | 0 | NA | 0 | NA |
| **N=1,000** | | | | | | | | |
| Balanced (1:1) | 0.01 | 5e-04 | 0 | NA | 0.0125 | 0.00181 | 0.015 | 0.0014 |
| Imbalanced (1:9) | 2.33 | 5e-04 | 0 (0.0025) | NA (0.012) | 0 | NA | 0 | NA |
| Extreme imbalanced (1:99) | 34.895 | 0.003 | 0 (1361.16) | NA (0.041) | 0 | NA | 0 | NA |
| **N=2,000** | | | | | | | | |
| Balanced (1:1) | 0.005 | 0.05025 | 0 (0.0050) | NA (0.050) | 0.0025 | 0.00107 | 0 | NA |
| Imbalanced (1:9) | 0.73 | 0.00025 | 0 | NA | 0.005 | 0.00156 | 0 | NA |
| Extreme imbalanced (1:99) | 20.85 | 0.002 | 0 (374.74) | NA (0.030) | 0.0075 | 0.0033 | 0.015 | 0.00528 |
| **N=5,000** | | | | | | | | |
| Balanced (1:1) | 0 | NA | 0 | NA | 0 | NA | 0 | NA |
| Imbalanced (1:9) | 0.0975 | 6e-04 | 0 (0.0025) | NA (0.0036) | 0.015 | 0.0011 | 0.0025 | 0.03013 |
| Extreme imbalanced (1:99) | 7.7875 | 0.0012 | 0 | NA | 0.05 | 0.00534 | 0.0325 | 0.00571 |
| **N=10,000** | | | | | | | | |
| Balanced (1:1) | 0 | NA | 0 | NA | 0 | NA | 0 | NA |
| Imbalanced (1:9) | 0.0325 | 0.001 | 0 | NA | 0.02 | 0.00068 | 0 | NA |
| Extreme imbalanced (1:99) | 2.515 | 0.001 | 0 | NA | 0.0725 | 0.00488 | 0.0325 | 0.00415 |

*For SAIGE, we excluded variants when SPA algorithm didn’t converge. The overall numbers of GWV and the corresponding median of MAF reported by SAIGE (regardless of the SPA convergence) are presented in parenthesis.

**Supplementary Table 4.** Mean and median of minor allele counts of the testing variants, stratified by SAIGE’s SPA algorithm convergence status.

|  | MAC | |
| --- | --- | --- |
|  | (SPA converged) | (SPA did not converge) |
| N=500 | | |
| Mean | 94.2 | 73.4 |
| Median | 28 | 19 |
| N=1,000 | | |
| Mean | 164.5 | 148.9 |
| Median | 42 | 38 |
| N=2,000 | | |
| Mean | 297.5 | 296.1 |
| Median | 66 | 74 |

**Supplementary Table 5.** Variants identified by Tractor with suggestive significance (ancestry specific p-value $\leq{1*10}^{-5}$).

| **CHROM** | **POS** | **REF** | **ALT** | **NAA MAF** | **EUR MAF** | **NAA p-value** | **EUR p-value** |
| --- | --- | --- | --- | --- | --- | --- | --- |
| 2 | 158,794,343 | G | A | 0.14 | 0.09 | 6.54e-06 | 0.96 |
| 3 | 153,553,199 | TTA | T | 0.31 | 0.35 | 5.87e-06 | 0.68 |
| 3 | 153,571,604 | C | T | 0.32 | 0.31 | 9.88e-06 | 0.90 |

**Supplementary Table 6. The computational times of the testing methods.** The experiments were conducted on a system with Debian GNU/Linux 10 as its operating system. The machine's architecture is x86_64, with CPU op-modes for 64-bit. It's powered by an 8-core Intel(R) Xeon(R) CPU E5-2620 v4, operating at a base frequency of 2.10GHz, and observed frequencies of 1200.392 MHz. For each job executed on this setup, a memory allocation of 20GB was designated.

|  | GMMAT | SAIGE | Tractor |
| --- | --- | --- | --- |
| Mean (s) | 121.37 | 98.73 | 926.65 |
| Median (s) | 119.74 | 89.00 | 911.00 |

**Supplementary Figure 1.** Quantile-Quantile plots for Tractor, stratified by MAF (common (MAF≥0.01) vs. rare (MAF<0.01) variants) and by case-control ratio.


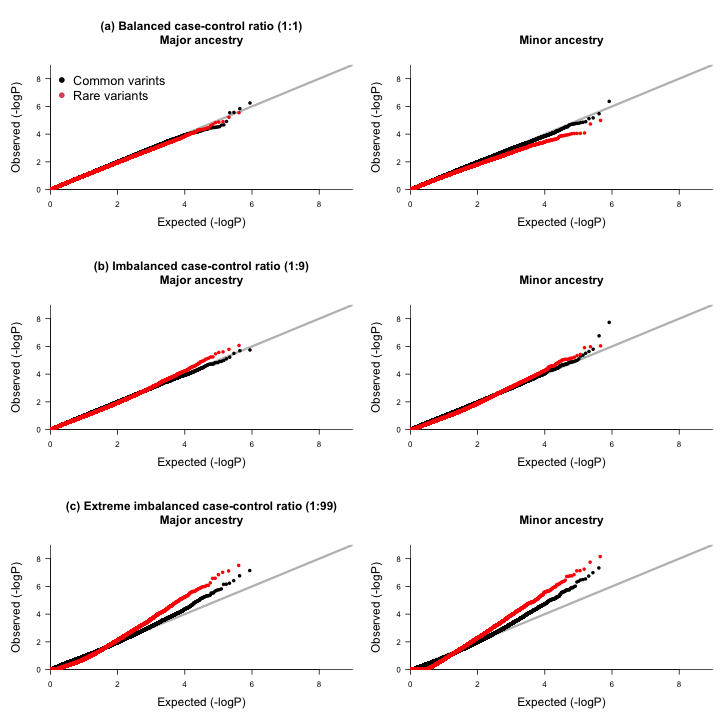
**Supplementary Figure 2.** Power calculation under varying sample sizes in HAPNEST (N=500/1,000/2,000/5,000/10,000, and 19,234) and $\beta_{NAA}$= 0.5, 1, 2, and 3 under the scenario of ultra-rare, rare, uncommon, common causal variant respectively. The significance threshold of p-value is set at genome-wide significance (p$\leq{5*10}^{-8}$). The causal ancestry, i.e. the ancestry with non-zero effect size, is NAA. The categories of variant MAF are defined as follows: "ultra-rare" (MAF<0.001), ****"rare" (0.001≤MAF<0.01), "uncommon" (0.01≤MAF<0.05), and "common" (MAF≥0.05).

**Supplementary Figure 3.** Impact of heterogeneity of the true effect sizes when there are two causal variants harboring within the two local ancestries (i.e. NAA and EUR). The effect size of the variant associated with the minor ancestry ($\beta_{NAA}$) ranges from -0.5 to 0.65 by 0.05, whereas the effect size of the variant associated with the major ancestry ($\beta_{EUR}$) is fixed at 0.15.
